# Characterization of the Anti-HCV Activities of the New Cyclophilin Inhibitor STG-175

**DOI:** 10.1371/journal.pone.0152036

**Published:** 2016-04-22

**Authors:** Philippe A. Gallay, Udayan Chatterji, Michael D. Bobardt, Zhengyu Long, Shengli Zhang, Zhuang Su

**Affiliations:** 1 Department of Immunology & Microbial Science, The Scripps Research Institute, La Jolla, California, 92037, United States of America; 2 S & T Global, Inc., 470 Wildwood Ave, Unit 3, Woburn, MA, 01801, United States of America; University of Padua, ITALY

## Abstract

Shortened current direct-acting antiviral (DAA) therapies while less expensive, have not provided satisfactory efficacy in naïve cirrhotics, treatment experienced non-cirrhotics or even genotype-3 (GT3)-infected patients. Since DAA regimens consist of the same classes of inhibitors—NS5A (NS5Ai) and NS5B (NS5Bi) +/- NS3 (NS3i) inhibitors—it is likely that their costs will be high and will provide similar degrees of protection. Integrating drugs with distinct mechanisms of action (MoA) into DAA regimens could provide the solution for shortening the period of treatment. One such class of agents is the cyclophilin inhibitors (CypI), which has shown efficacy in patients. Resistance-associated variants persist for years post-treatment in patients exposed to NS5Ai or NS5Bi who fail to achieve a sustained virologic response, impairing their chance for cure on retreatment with existing DAA combinations. Because of their high barrier to resistance, CypI may be particularly useful as a rescue therapy for patients who have relapsed with DAA resistance-associated variants. In this study, we analyzed the anti-HCV properties of the novel cyclosporine A (CsA) derivate—STG-175. The non-immunosuppressive STG-175 possesses a high (EC_50_ 11.5–38.9 nM) multi-genotypic (GT1a to 4a) anti-HCV activity. STG-175 clears cells from HCV since no viral replication rebound was observed after cessation of drug treatment. It presents a higher barrier to resistance than other CypI or selected DAAs. HCV variants, which emerged under STG-175 pressure, are only ~2-fold resistant to the drug. No cross-resistance was observed with DAAs STG-175 was efficacious against DAA-resistant HCV variants. Drug combination studies revealed that STG-175 provides additive and synergistic effects against GT1a to 4a. STG-175 inhibits the infection of HCV, HIV-1 and HBV in mono-, dual- and triple-infection settings. Altogether these results suggest that the new CypI STG-175 represents an attractive drug partner for IFN-free DAA regimens for the treatment of HCV and co-infections.

## Introduction

Nearly 200 million people are infected with hepatitis C virus (HCV) and chronic hepatitis C is a leading cause of liver diseases [1]. Four million people are newly infected every year [2–3]. In the developed world, two-third of transplant and liver cancer cases are caused by chronic hepatitis C [4]. Until recently, an IFNα/ribavirin regimen had a success rate of ~80% in GT2- and GT3-infected patients, of ~50% in GT1-infected patients, and was associated with serious side effects [5–9]. Therefore, there was an urgent necessity for the identification of anti-HCV agents in order to provide substitute regimens for IFN/RBV therapies. Importantly, DAAs such as NS3i, NS5Ai and NS5Bi have been identified [10], and most importantly, several of them are currently included in safe and efficacious IFN/RBV-free regimens. Yet, these DAA IFN-free anti-HCV therapies are expensive [11]. One approach to reducing the cost of hepatitis C treatment is to shorten the duration of the drug treatment. However, shortening therapy from 24 to 12 weeks to reduce costs did not provide satisfactory efficacy in naïve cirrhotics, treatment experienced non-cirrhotics or even GT3-infected patients [12–13]. An alternative approach for reducing the cost of hepatitis C treatment is to identify new drug combinations that would provide safety, efficacy and truncated treatment option. Since the new IFN-free regimens consist mainly of combinations of the same classes of inhibitors—NS5Ai, NS5Bi and NS3i—it is likely that their respective costs will also be high and that they will provide similar degrees of protection in short or long therapies. Moreover, the possibility of drug resistance and unexpected side effects cannot yet be ruled out [14]. On the other hand, the possibility of integrating new anti-HCV agents with distinct MoAs into current IFN-free DAA regimens could provide the solution to efficiently shorten the period of treatment. One attractive class of anti-HCV agents, with a MoA distinct from the DAAs -NS5Ai, NS5Bi and NS3i,—is the CypI. CypI, which target a host protein–cyclophilin A (CypA),–rather than a viral protein, showed high potency in multiple clinical studies. In particular, the CypI alisporivir (ALV) provided high safety and efficacy when combined with IFN or as IFN-free regimen in GT2 and GT3-infected patients [15–20]. We showed that a combination of CypI and NS5Ai, NS5Bi or NS3i provides additive to synergistic effects on GT1 to 4 and no cross-resistance [21]. We also showed that a combination of CypI with NS5Bi is promising against GT3 [21]. Thus, CypI can be used in combination with DAAs in patients to attempt shortening current costly therapies. Importantly, resistance-associated variants persist for several years post-treatment in patients exposed to NS5Ai or NS5Bi who fail to achieve an SVR [14, 17, 22–23], possibly impairing their chance for cure on retreatment with existing DAA combinations. Because of their high barrier to resistance, CypI may be particularly useful in combination with NS5Bi as a rescue therapy for patients who relapse with DAA resistance-associated variants. In this study, we analyzed the *in vitro* anti-HCV properties of a novel CypI called STG-175.

## Material and Methods

### Compounds

The preparation of STG-175 (molecular weight 1336,83 Da) was based on the US Patent Application Publication No.: US 2013/0210704 A1, Zhuang Su et al., Novel Cyclosporin Derivatives and Uses Thereof, Aug. 15, 2013. The NS5Ai daclatasvir (Bristol Myers Squibb), the NS5Bi sofosbuvir (Gilead) and the NS3i boceprevir (Merck) and telaprevir (Vertex) were obtained from MedChemexpress (Princeton, NJ 08540, USA). ALV (Debiopharm) was obtained from Acme Bioscience whereas CsA, ribavirin (RBV) and IFNα2a from Sigma.

### Anti-peptidyl-prolyl isomerase (PPIase) assay

Inhibition of CypA and CypD isomerase activities were quantified using a α-chymotrypsin-coupled assay adapted to a 96-well plate format [24–25]. Human recombinant CypA or CypD (Atgen) was dissolved to 10 nM in PPIases buffer (50 mM Hepes, 100 mM NaCl, 1 mg/ml bovine serum albumin, 1 mg/ml α-chymotrypsin; pH 8). Succinyl-AAPF-pNA peptide substrate (Sigma) was dissolved to 3.2 mM in LiCl/trifluoroethanol. Each CypI was prepared at 10 concentrations in DMSO, then diluted into CypA/D PPIase buffer to 0.05–1000 nM. Incubations were performed at 5°C. Ninety five μL of reaction mix was added to 5 μL peptide in 96-wells and OD405 nm measured at 6-sec intervals for 6 min using a Biotek Synergy H4 plate reader. Data were fitted with Graphpad Prism 6.0 to acquire first-order rate constants, which were calculated by subtracting the rate constant from uncatalyzed reactions (no CypA/D). The catalytic rate constants were plotted as a function of inhibitor concentration to obtain IC_50_s.

### *In vitro* assay for immunosuppression

IL-2 promoter activation upon T-cell stimulation was determined using an engineered Jurkat T-cell line, which expresses betagalactosidase when exposed to chemical non-specific factors like phorbol-13-myristate acetate (PMA) and phytohematogglutinin (PHA) as described previously [26–27]. DMSO or STG-175 were added together with PMA (10 ng/mL) to 1 million of Jurkat cells (triplicates). Betagalactosidase activity in cell lysates was quantified by fluorescence (460 nm) after 24 h.

### Replicon cell lines and antiviral and cytotoxic assays

The anti-HCV activity of STG-175 was conducted using a panel of luciferase reporter replicon cell lines including GT1a, GT1b, GT2a, GT3a and GT4a as we described previously [21]. The GT1a subgenomic *renilla* luciferase reporter replicon H77 RLucP (7) was supplied by Dr. Delaney [28]. The GT1b subgenomic *firefly* luciferase reporter replicon pFK-I389/NS3-3’ [29] was supplied by Dr. Bartenschlager. The GT1B subgenomic NS3, NS5A and NS5B mutants were created via homologous recombination using the In-Fusion HD Cloning kit (Clontech) as we described previously [21]. The GT2a genomic luciferase reporter replicon Luc-Neo-JFH-1 was generated as previously [30]. The plasmid pFK-Luc-JFH1 was supplied from Drs. Wakita and Pietschmann [31–32] and the XbaI site in the *firefly* luciferase gene and the NotI site in the EMCV IRES were used to take the Luciferase/Ubiquitin-NPT II (the neomycin phosphotransferase II gene) fusion cassette out of pFK389ILuc-Neo (wild-type replicon from GT1b) and inserted into the pFK-Luc-JFH1 plasmid, generating full-length Luc-Neo-JFH-1 [30]. The GT3a subgenomic *firefly* luciferase reporter replicon S52/SG-Feo (AI) and the GT4a subgenomic *firefly* luciferase reporter replicon ED43/SG-Feo [33] were supplied by Drs. Rice and Bukh. Replicons were stably expressed in Huh7.5 or Huh7.5.1 cells under G418 selection. On Day 1, cells (5,000) were plated into 96-well plates in complete DMEM containing 10% FCS. On Day 2, increasing drug concentrations were added to wells. On Day 5, 72 h post-drug addition, medium was removed and replaced with 100 μL of fresh medium. Hundred μL of the fluorogenic, cell-permeant peptide substrate glycyl-phenylalanyl-amino fluorocoumarin was added to wells as per manufacturer’s recommendation (Promega Corp, Madison, WI). The CellTiter-Fluor™ Cell Viability Assay is a non-lytic fluorescence assay, which measures the number of live cells. Cells were incubated at 37°C for 1 h and assayed on a fluorescence plate reader (Biotek, Synergy H4) at an excitation of 390 nm and emission of 505 nm. Medium was removed, cells washed and lysed in 20 μL of Cell Culture Lysis Reagent. Luciferase activity was determined using the Luciferase Assay System (Promega) in a Berthold luminometer. The alamarBlue® assay (Thermo Fischer Scientific) was used to measure CC_50s_ for STG-175 on replicon cell lines. When cells are alive they maintain a reducing environment within the cytosol of the cell. Resazurin, the active ingredient of alamarBlue® reagent, is a non-toxic, cell permeable compound that is blue in color and virtually non-fluorescent. Upon entering cells, resazurin is reduced to resorufin, a compound that is red in color and highly fluorescent. Viable cells continuously convert resazurin to resorufin, increasing the overall fluorescence and color of the media surrounding cells.

### Drug combination studies

Drugs were tested in pairs in 7 concentrations around the calculated EC_50_ in each replicon cell line. Cell viability and reporter assays were carried out as we described previously [21] in 5 replicates and normalized data was used to measure the additive, synergistic and antagonistic effect between drug combinations using the MacSynergyII program. This program is based on the Bliss independence model that is defined by the equation Exy = Ex + Ey − (/Ex × Ey), where (Exy) is the additive effect of drugs x and y as predicted by their individual effects (Ex and Ey) [34].

### Cross-resistance studies

The GT1b subgenomic firefly luciferase reporter replicon pFK-I389/NS3–3 was provided by R. Bartenschlager. The GT1B subgenomic NS3, NS5A and NS5B mutants were created via homologous recombination using the In-Fusion HD Cloning kit (Clontech) as we described previously [21]. Specifically, we generated the following DAA-resistant Con1 GT1b replicons: i) the protease inhibitor-resistant R155Q/A156T NS3 replicon; ii) the daclastavir-resistant L31V NS5A replicon; iii) the polymerase inhibitor-resistant S282T NS5B replicon; and iv) the STG-175-resistant D320E and D320E/Y321N NS5A replicons (see below). The *in vitro* transcription of wild-type and mutant Con1 RNA was accomplished using the T7 MEGAscript kit (Ambion) by following the manufacturer’s instructions. *In vitro*-transcribed RNAs were introduced into Huh7.5.1 cells by electroporation. Trypsinized cells were washed twice and resuspended in phosphate-buffered saline (PBS) (calcium-free and magnesium-free) at 1x 10^7^ cells per ml. Ten micrograms of RNA for each mutant was mixed with 0.4 mL of cells in a 4-mm cuvette, and a Bio-Rad Gene Pulser system was used to deliver a single pulse at 0.27 kV, 100 ohms, and 960 μF. Cells were then plated in 12-well dishes. RNA transfection efficiency and HCV subgenomic replication were assessed by reverse transcription-quantitative PCR (RT-qPCR) and presented as genome equivalents (GE) per microgram of total RNA as described previously [35]. We measured by RT-qPCR the replication of wild-type, STG-175-, NS5Ai-, NS3i- and NS5Bi-resistant mutants in the presence of increasing concentrations of SGT-175, daclatasvir, telaprevir, boceprevir and sofosbuvir over a period of 8 days.

### Clearance and replication rebound studies

Huh-7 cell expressing subgenomic Con1 (GT1b) replicon were seeded in a 10 cm dish at a density of 3 × 10^5^ cells per culture flask in complete DMEM (without G418) with or without STG-175 or selected drugs. Cells were grown until they reached 90% confluence, trypsinized and re-seeded at the same cell concentration in a new 10-cm dish with the same drug concentration. Cells were passaged 7 times in the presence of drugs. Then drugs were removed to allow viral replication rebound to occur. At each passage, sample of cells were collected, RNA extracted according to the manufacturer's instructions and analyzed by RT-PCR for HCV RNA levels.

### Emergence kinetics of drug resistance

Huh-7 cell expressing subgenomic Con1 (GT1b) replicon were seeded in a 10 cm dish at a density of 3 × 10^5^ cells per culture flask in complete DMEM (with G418) with STG-175 or selected drugs. Cells were grown until they reached 90% confluence, trypsinized and re-seeded at the same cell concentration in a new 10-cm dish with the same drug concentration. Cells were passaged 22 times in the presence of drugs. At each passage, sample of cells were collected, RNA extracted according to the manufacturer's instructions and analyzed by RT-PCR for HCV RNA levels.

### Selection of resistant replicon cell lines

Huh-7 cells expressing subgenomic Con1 (GT1b) replicon were incubated with STG-175 and 100 μg/ml of G418 in 10 cm CellBIND™ plates (Corning). Cells were split every 3 days at 1:6 and G418 concentration was increased sequentially to 200 and 300 μg/mL. Most of the cells were dying 3 weeks after drug incubation. At this point, medium was changed every 3–4 days until emergence of resistant clones. Clones were individually transferred to 24-well CellBIND using cloning disks. Several clones did survive after the transfer process and were expanded to 6-well plates. Each clone was split in 2 identical plates and was separately maintained under 0.5 or 1.0 μM STG-175 and 100 μg/mL of G418. Once the colonies were established, G418 concentration was increased to 300 μg/mL. Ultimately, colonies growing even in the presence of the drug were lysed, RNA extracted and NS3-NS5B region sequenced. Briefly, total RNA was prepared using the RNeasy Plus Mini Kit as per manufacturer’s recommendation. Two hundred ng of total RNA was used for cDNA production using primers specific to the NS3-NS5B region or oligo(dT)_18_ using AccuScript High Fidelity 1^st^ Strand cDNA Synthesis Kit (Stratagene). Amplicons were generated using primers defining the sequence at its 5’ and 3’ ends. The amplicons were gel purified, quantitated and sequenced using primers located internally in NS5A or NS5B reading to the 5’ and 3’ ends. Sequences were assembled and analyzed using the ClustalW algorithm within the MacVector program. The sequencing was conducted twice to ensure the authenticity of the nucleotide substitutions.

### Analysis of STG-175-resistant variants

D320E and D320E/Y321N NS5A substitutions were introduced into wild-type Con1 GT1 replicon as we described previously [21]. Con1-NS5A-D320E, and Con1-NS5A-D320E/Y321N were generated using the following forward and reverse phosphorylated (p) primer sets, respectively: (p)GGGCACGCCCGGAATACAACCCTCCACTGT and (p)ATATGGGCATCGCTCGAGGGAATTTCCTGG, and (p)GAGAACAACCCTCCACT GTTAGAGTCCTGGAAGGA and (p)CGGGCGTGCCCATATGGGCAT CGCTCGAGGGAATT. PCR products were circularized with Quick T4 DNA ligase (NEB) for 5 min and transformed into NEB 10-beta competent cells. The NS5A gene was sequenced to confirm the introduced substitutions.

### Mono- and multi-infection assays

For mono-infections: a) human hepatoma Huh7.5.1 cells were infected with HCV (JFH-1) and viral replication was quantified by HCV core ELISA (Ortho HCV antigen ELISA kit; Ortho Clinical Diagnostics, Waco Chemicals, Inc. or HCV Core ELISA, XpressBio); b) sodium-taurocholate co-transporting polypeptide (NTCP)-positive Huh7 cells were infected with hepatitis B virus (HBV) AD38 (produced from HepAD38 cells) and viral replication was quantified by HBV HBeAg ELISA; and c) human (peripheral blood monocytic cells (PBMCs) were infected with HIV-1 (JR-CSF) and viral replication was quantified by HIV-1 capsid/p24 ELISA. PBMCs were isolated as described previously [36]. The Scripps Research Institute Normal Blood Donor Service (TSRI NBDS) provides TSRI investigators who have Human Subjects Committee-approved protocols with a source of normal blood for their research. Donors are assured of a controlled clinical setting for their blood to be drawn by licensed phlebotomists, and investigators are assured that the donors whose specimens they obtain through the service have been screened upon entry into the program and annually thereafter for a CBC, Hepatitis B and C and HIV-1. Hemoglobin determinations at every donation protect the donor from phlebotomy-induced anemia. The donor pool also provides investigators with a mix of gender and minority subjects, and recruitment is ongoing for underrepresented minorities. At the present time, the NBDS has 320 active normal blood donors enrolled. Use of the Normal Blood Donor Service is considered “human subjects” research and each investigator who wants to use the service must submit a protocol to the IRB for review and approval. Dr. Gallay has current IRB approval for the human blood obtained for research described in this manuscript. Protocol No: IRB-15-6552. Full name of the IRB is "Scripps IRB". The IRB entitled "Evaluation of the antiviral activities of CypI on the hepatitis/HIV-1 co-infection" specifically approved the use of blood donor in this study. Consent from blood donors was obtained verbally. The authors have no access to identifying donor information for these samples. The TSRI Normal Blood Donor Program is an IRB-approved protocol that uses two written consent forms: the first for screening donors when they join the program and each year thereafter; and the second for each blood donation. Because donors may give up to a unit of blood to be shared among several investigators, they are presented with a brief description of each investigator’s planned use of their blood- this part is not a consent form, but rather an information sheet, allowing them to opt out of one or more research uses of their blood, should they choose to do so. The ethics committees/IRBs approve this consent procedure. The primary HIV-1 isolate JR-CSF virus was obtained through the NIH AIDS Research and Reference Reagent Program. JR-CSF was amplified in PBMC (5 x 10^6^ cells), and infectious particles collected 2–3 weeks post-infection and quantified by HIV-1 p24 ELISA (HIV1: Alliance HIV-1 p24 ELISA Kit from PerkinElmer). *Mono-infections*: HCV: Huh7.5.1 cells (500,000) were incubated 3 h with JFH-1 (100 ng of core), washed and grown for 3 weeks (triplicates). Aliquots of supernatants were collected every 3 days, filtered and stored until HCV measurement by core ELISA. HBV: NTCP-Huh7 cells (500,000) were exposed to 100 ng of core of AD38 for 3 h, washed and cultured for 3 weeks in flasks (triplicates). Aliquots of cell culture supernatants were collected every 3 days, filtered to remove cell debris and frozen until HBV quantification by core ELISA. NTCP-Huh7 cells were created by stably transfecting the CMV-C-terminal tagged Myc-Flag hNTCP (Origene) into Huh7 cells under G418 selection. HIV-1: Activated PBMCs (500,000) were exposed to 1 ng of p24 of JR-CSF for 3 h, washed and cultured for 3 weeks in flasks (triplicates). Aliquots of cell culture supernatants were collected every 3 days, filtered to remove cell debris and frozen until HIV-1 quantification by p24 ELISA. *Co-infections/Concurrent infections*: Mixed PBMCs, Huh7.5.1 cells and NTCP-Huh7 cells in a single flask were exposed for 3 h to HCV, HBV and HIV-1 and viral replication monitored over a period of 3 weeks as described above. For drug treatments, STG-175 was added to cells either i) together with viruses or ii) 3 days post-infection and then every 3 days for a period of 12 days.

## Results

### Inhibition of the isomerase activity of CypA and CypD by STG-175

**Fig 1:**
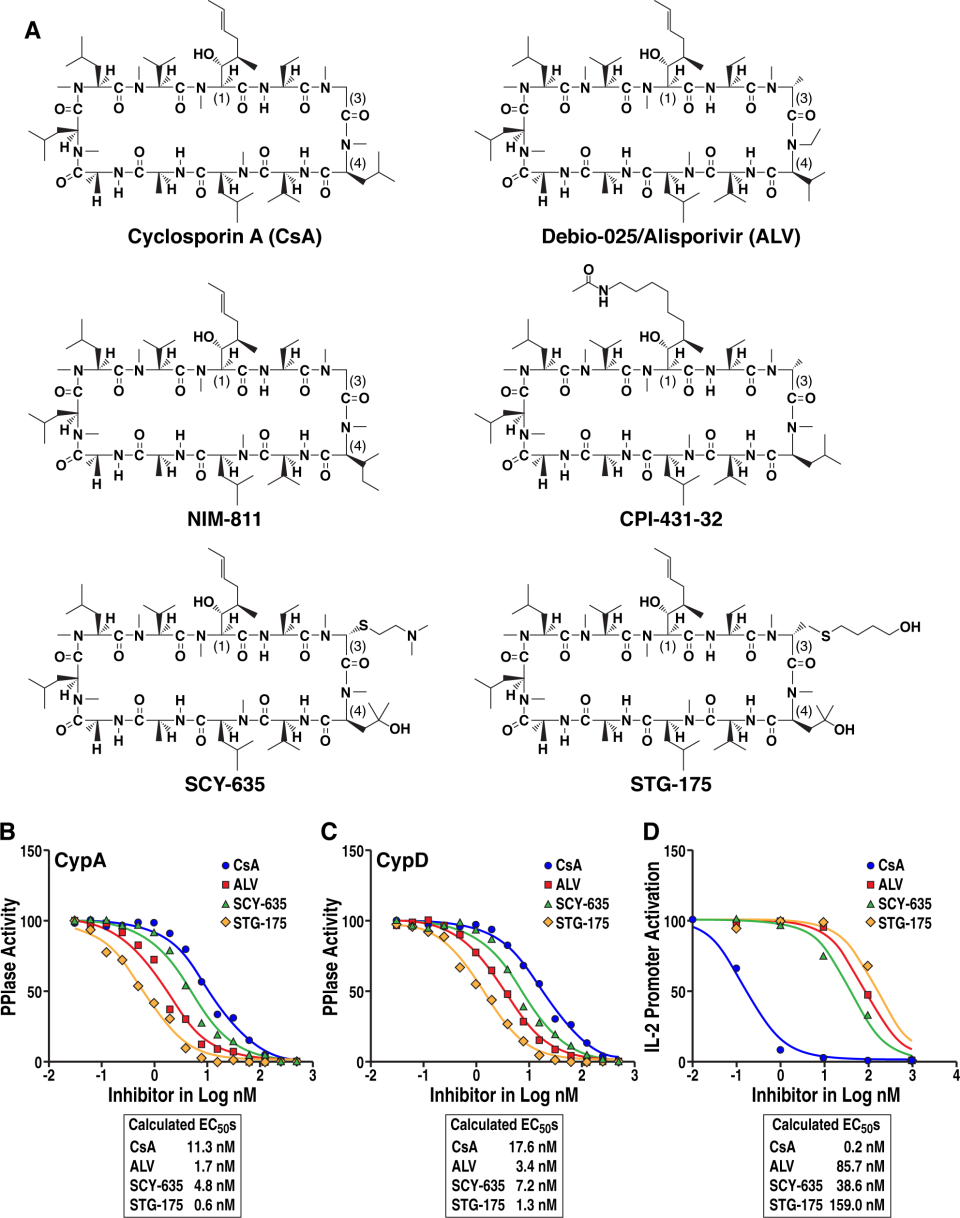
Anti-PPIase and immunosuppressive activities of STG-175. A. Structures of cyclophilin inhibitors. Inhibition of CypA (B) and CypD (C) isomerase activities were assessed using the α-chymotrypsin-coupled assay [24]. Each test compound was prepared at 10 concentrations (0.05–1000 nM). All solutions were equilibrated, and reactions conducted at 5°C. Reactions were initiated by mixing 95 μL reaction mix with 5 μL of the peptide to be cleaved and measuring OD405 nm at 6-sec intervals for 6 min. Data were fitted with Graphpad Prism 6.0 to obtain first-order rate constants. Enzyme catalyzed rate constants were calculated by subtracting the rate constant from uncatalyzed reactions (no CypA/D), and catalytic rate constants plotted as a function of inhibitor concentration to obtain IC_50_s. Data are representative of two independent experiments. D. IL-2 promoter activation upon T-cell stimulation was determined using an engineered Jurkat T-cell line, which expresses betagalactosidase. Jurkat cells (1 million) were activated with PMA (10 ng/mL). DMSO, STG-175, CsA, ALV or SCY-635 were added together with PMA and enzymatic activity in cell lysates quantified after 24 h. The Y-axis values are expressed as percentage of activity. Data expressed as EC_50_s are representative of two independent experiments.

We first analyzed the anti-PPIase activity of the CsA derivate STG-175 using the chymotrypsin-coupled CypA isomerase assay originally developed by Fischer et al. [24]. The inhibition of the isomerase activities of CypA and CypD were measured with 10 increasing concentrations of STG-175 to quantify IC_50_ values. We used as controls the CypI CsA, ALV and SCY-635. The structures of the cyclophilin inhibitors used in this study are presented in Fig 1A. For the neutralization of the isomerase activity of CypA, we calculated IC_50_ of 11.3, 1.7, 4.8 and 0.6 nM for CsA, ALV, SCY-635 and STG-175, respectively (Fig 1B). For the neutralization of the isomerase activity of CypD, we calculated IC_50_ of 17.6, 3.4, 7.1 and 1.3 nM for CsA, ALV, SCY-635 and STG-175, respectively (Fig 1C). The degree of neutralization was the following: STG-175 > ALV > SCY-635 > CsA. These results indicate that STG-175 possesses a high anti-PPIase activity, at least for two main members of the cyclophilin family–CypA and CypD.

### Immunosuppressive activity of STG-175

The immunosuppressive activity of CsA is mediated by the initial formation of a complex between CsA, CypA and calcineurin. The formation of this trimolecular complex inhibits T cell proliferation by blocking the signaling pathway leading to IL-2 production upon T-cell stimulation. The immunosuppressive activity of STG-175 was evaluated by inhibition of the IL-2 production in Jurkat cells. We calculated IC_50_ of 0.2, 85.7, 38.6 and 159 nM for CsA, ALV, SCY-635 and STG-175, respectively (Fig 1D). The finding that STG-175 exhibits approximately 1000 times less immunosuppressive activity than CsA suggests that the CypI STG-175 should not induce significant undesirable inhibition of T cell proliferation *in vivo*.

### Anti-HCV activities of STG-175

**Fig 2:**
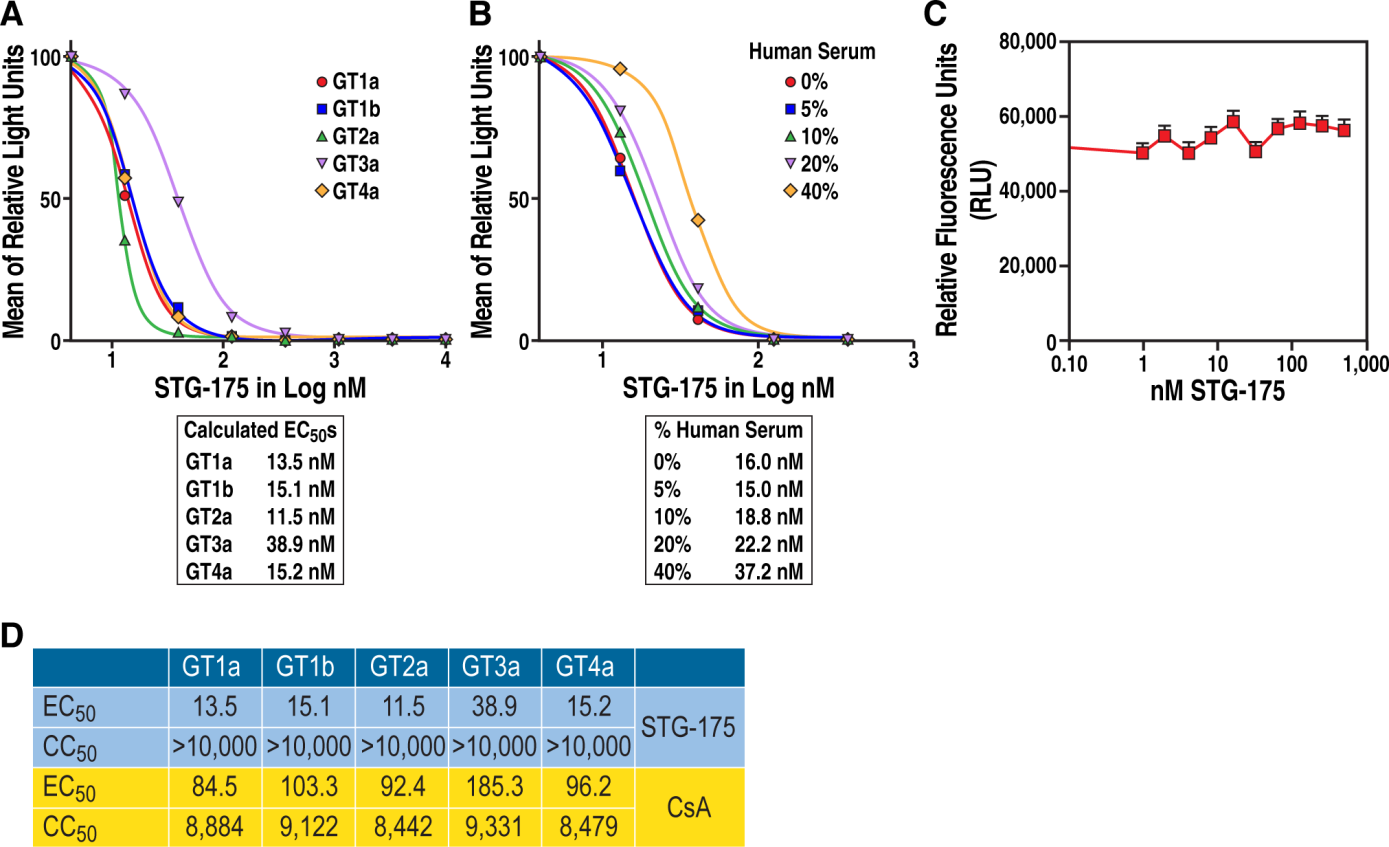
Anti-HCV activities of STG-175 among GTs. A. Replicon-containing cells (5,000) were exposed to increasing concentrations of the indicated drugs. Seventy-two hours post-drug addition, the fluorogenic, cell-permeant peptide substrate glycyl-phenylalanyl-amino fluorocoumarin was added to measure the number of live cells. Cells were incubated at 37°C for 1 h and assayed for fluorescence at an excitation of 390 nm and emission of 505 nm. Luciferase activity was determined using the Luciferase Assay System in a Berthold luminometer. B. A similar experiment was conducted with the GT1b replicon cell line in 0, 5, 10, 20 and 40% of human serum. Data are expressed as mean of relative light units and calculated EC_50_s are presented. Data are representative of two independent experiments. C. As illustration of the lack of cellular cytotoxicity of STG-175, cell viability of the GT2a replicon cell line toward increasing doses of STG-175 was analyzed by the alamarBlue® cell viability assay. Data are representative of two independent experiments. D. EC_50s_ (concentration of 50% effective antiviral inhibition) and CC_50s_ (concentration of 50% cellular cytotoxicity) for STG-175 and CsA were quantified for each genotype replicon cell line.

We then evaluated the anti-HCV activities of STG-175 on GT1a, GT1b, GT2a, GT3a and GT4a replicons. We found that STG-175 inhibits at a nM range the replication of all GTs: GT1a (EC_50_ = 13.5 nM), GT1b (EC_50_ = 15.1 nM), GT2a (EC_50_ = 11.5 nM), GT3a (EC_50_ = 38.9 nM) and GT4a (EC_50_ = 15.2 nM) (Fig 2A). This demonstrates the effective multi-genotypic antiviral activity of STG-175. Importantly, the antiviral activity of STG-175 is only partly decreased in the presence of high concentration of human serum. Specifically, we calculated for GT1b, EC_50_ of 16, 15, 18.8, 22.2 and 37.2 nM in 0, 5, 10, 20 and 40% of human serum, respectively (Fig 2B). STG-175 does not mediate its antiviral activity by influencing the viability of the replicon cell lines (Fig 2C and 2D).

### Efficacy of STG-175 on HCV clearance and replication rebound

**Fig 3:**
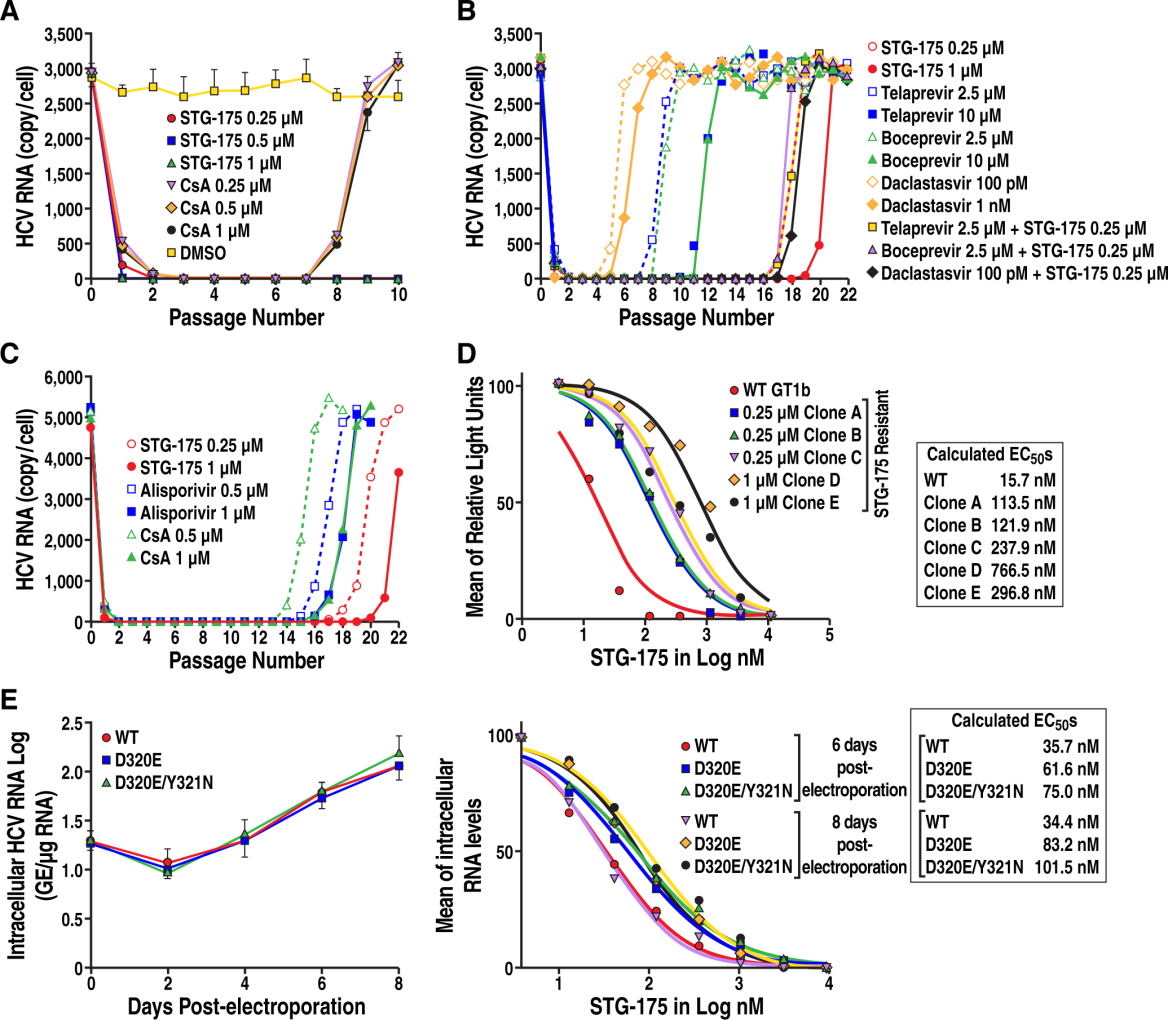
Replicon clearance, replication rebound and resistance analyses. A. *Clearance and replication rebound*: Con1 replicon Huh-7 cells were passaged 7 times in the absence or presence of STG-175 or CsA (0.25, 0.5 and 1 μM). Then drugs were removed to allow viral replication rebound to occur. At each passage, samples of cells were collected, RNA extracted and analyzed by RT-PCR for HCV RNA levels. B. *Emergence of resistance*: Con1 replicon Huh-7 cells were passaged 22 times in the absence or presence of STG-175 or selected DAAs. At each passage, sample of cells were collected, RNA extracted and analyzed by RT-PCR for HCV RNA levels. C. Same as B except that selected CypI were compared–STG-175, ALV and CsA. Data are expressed as number of HCV RNA copies per cell. Data are representative of two independent experiments. D. *Degree of resistance of STG-175-resistant colonies*: STG-175-resistant colonies, which emerged under drug selection, were analyzed for their resistance to increasing concentrations of STG-175. Data are expressed as mean of relative light units and calculated EC_50_s are presented. Data are representative of two independent experiments. E. *Degree of resistance of D320E and Y321N Con1 variants*: Left panel. Wild-type (WT), D320E and D320/Y321N NS5A Con1 RNA were electroporated into Huh7.5.1 cells and replication monitored over 8 days by quantifying the levels of intracellular HCV RNA. Right panel. Same as just above except that increasing concentrations of STG-175 were added at the time of the electroporation. Data were calculated as log of genome equivalents (GE)/μg of total RNA and the RNA amount was normalized to a range of 0–100% in order to calculate the EC_50_. Results are representative of two independent experiments.

Given that anti-HCV treatment involves weeks or months of drug administration, we examined the effect of an extended STG-175 treatment on HCV replication. For viral clearance, GT1b Con1 sub-genomic replicon cells were grown in the presence of the increasing concentrations of STG-175, CsA or DMSO for 7 successive passages. At each passage, fresh drug was added. After 7 passages, drugs were no longer added, but cells were further passaged 3 times (total of 10 passages) for viral replication rebound analysis. At each passage, a sample of cells was taken to quantify HCV RNA levels by RT-qPCR. After the first passage in the presence of STG-175 or CsA, a profound decrease in HCV replication was observed (Fig 3A). The low-to-no viral replication was maintained until passage 7. Importantly, after the drug removal after 7 passages, no replication rebound was observed for replicons grown under STG-175 pressure—0.25, 0.5 or 1 μM—suggesting clearance of the cells from the replicon. In sharp contrast, replication rebound was observed for the replicon grown under CsA pressure—0.25, 0.5 or 1 μM–suggesting that CsA was unable to clear the replicon from the cells after 7 successive passages under drug pressure (Fig 3A). We obtained similar data for GT2a and GT3a (data not shown).

### Emergence kinetics of STG-175 resistance

We then examined the development of resistance to STG-175. GT1b Con1 sub-genomic replicon cells were grown in the presence of STG-175 (0.25 and 1 μM), telaprevir (2.5 and 10 μM), boceprevir (2.5 and 10 μM), daclatasvir 100 pM and 1 nM) or a combination of STG-175 (0.25 μM) with either telaprevir (2.5 μM), boceprevir (2.5 μM) or daclatasvir (100 pM) for 22 successive passages. At each passage, fresh drug was added and a sample of cells was taken to quantify HCV RNA levels by RT-qPCR. STG-175 resistance only started at passage 16 and 19 for 0.25 and 1 μM drug pressure, respectively (Fig 3B). ALV resistance began at passage 14 and 16 for 0.25 and 1 μM drug pressure, respectively, while CsA resistance began at passage 13 and 15 for 0.25 and 1 μM drug pressure, respectively. These results indicate that STG-175 offers a higher barrier to resistance than CsA and a comparable barrier to HCV resistance to ALV. Telaprevir resistance began at passage 7 and 10 for 2.5 and 10 μM, respectively, whereas boceprevir resistance commenced at passage 7 and 10 for 2.5 and 10 μM, respectively. Daclatasvir resistance started at passage 4 and 5 for 100 pM and 1 nM, respectively. Together these data suggest that the CypI STG-175 provides a high barrier to resistance compared to DAAs such as protease (telaprevir and boceprevir) and NS5A (daclatasvir) inhibitors. Importantly, when we combined STG-175 with telaprevir, boceprevir or daclatasvir, the development of HCV resistance to protease and NS5A inhibitors was greatly delayed– 16 passages instead of 6 for telaprevir, 16 instead of 7 for boceprevir, and 17 instead of 4 for daclatasvir (Fig 3B). Thus, these data strongly suggest that combining the CypI STG-175 with DAAs would greatly enhance the barrier to HCV resistance. We also compared the emergence of resistance between several CypI including STG-175, ALV and CsA. At 0.25 μM of drug selection, resistant variants emerged at passage 14, 15 and 18 for CsA, ALV and STG-175, respectively (Fig 3C). At 1 μM of drug selection, resistant variants emerged at passage 16, 17 and 21 for CsA, ALV and STG-175, respectively (Fig 3C). These data indicate that STG-175 offers the best barrier to resistance among the tested CypI.

### Analysis of STG-175-resistant HCV variants

**Fig 4:**
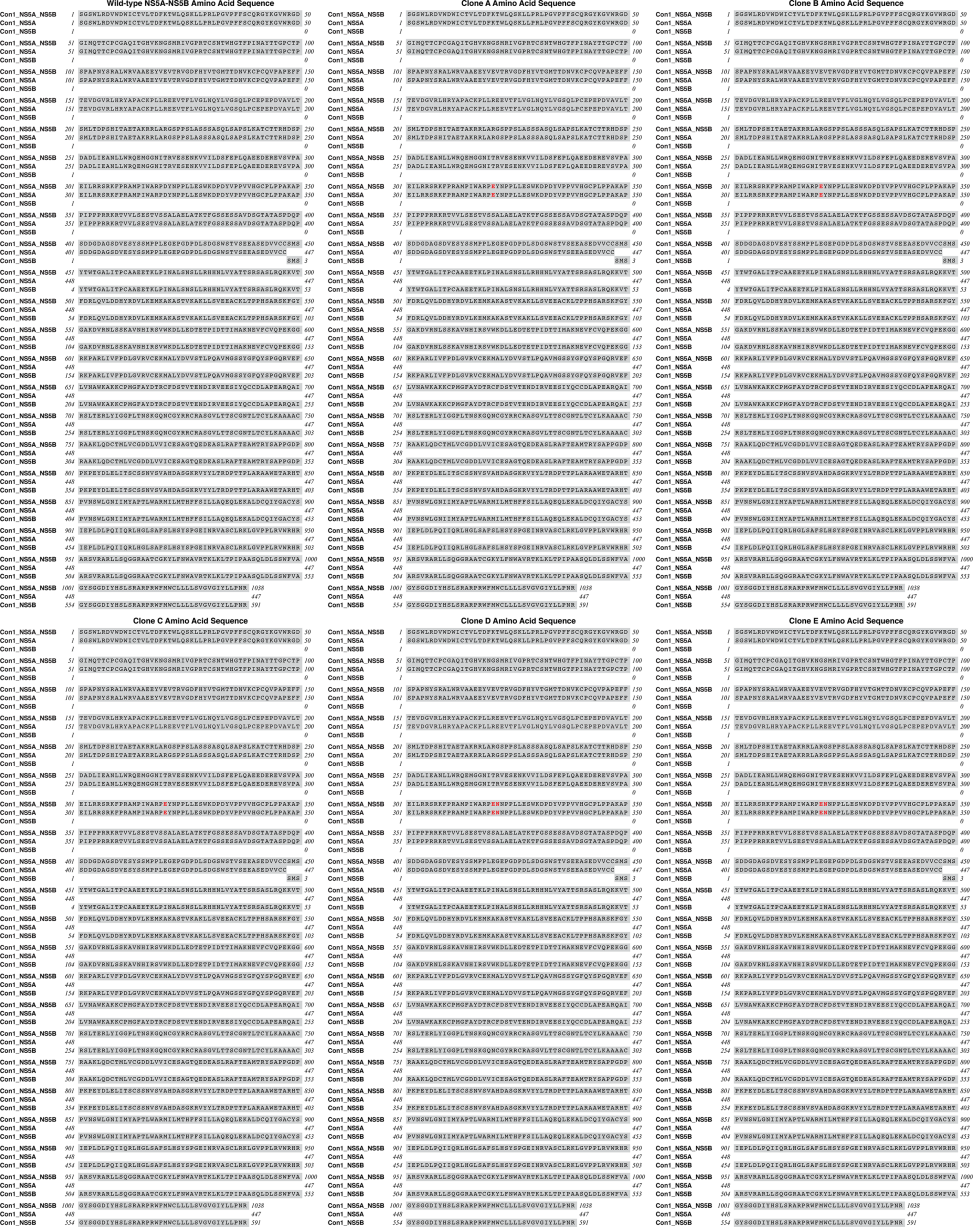
Amino acid sequences of NS5A-NS5B region of wild-type Con1 as well as NS5A-NS5B region of clones A to E.

We then sequenced the NS3-NS5B region of the HCV variants (colonies), which emerged after multiple passages under STG-175 selection. All five STG-175-resistant variants, which we selected, contain mutations in NS5A, but not in other regions of NS3-NS5B. The three variants, which grew in the presence of 0.25 μM of STG-175, developed a single mutation in NS5A –D320E, whereas the two variants, which grew in the presence of 1 μM of STG-175, developed two neighbor mutations in NS5A –D320E and Y321N (Fig 4). The five STG-175-resistant variants (5 colonies A to E) were then retested for STG-175 resistance by exposing them to increasing concentrations of STG-175 as we described previously [21]. We calculated EC_50_ of 113.5 (colony A), 121.9 (colony B) and 237.9 nM (colony C) for the three D320E NS5A variants, which emerged under 0.25 μM of STG-175 selection, and 766.5 (colony D) and 296.8 nM (colony E) for the two D320E/Y321N variants, which emerged under 1 μM of STG-175 selection, (Fig 3D). The enhancement of resistance to STG-175 of colonies A, B and C (D320E variants) were 7.2-, 7.8- and 15.1-fold increase compared to wild-type replicon, whereas the enhancement of resistance of colonies D and E (D320E/Y321N variants) were 48.8- and 18.9-fold increase compared to wild-type replicon.

To determine whether the D320E and Y321N substitutions truly render HCV resistant to STG-175, they were introduced into wild-type Con1 replicon as we described previously [21]. Introduction of the substitutions was verified by sequencing. *In vitro*-transcribed wild-type, D320E and D320E/Y321N HCV RNAs were then electroporated into Huh7.5.1 cells and viral replication assessed by RT-qPCR and presented as GE per microgram of total RNA as we described previously [35]. We found that the substitutions do not significantly alter the viral growth of the replicons since the variants replicate at wild-type levels (Fig 3E, left panel). We then analyzed the replication of wild-type, D320E and D320E/Y321N in the presence of increasing concentrations of SGT-175 over a period of 8 days. We calculated EC_50_ for the three replicons at day 6 and 8 post-electroporation. At day 6, the D320E substitution renders the replicon (EC_50_ of 61.6 nM) 1.7-fold more resistant to STG-175 than wild-type replicon (EC_50_ of 35.7 nM) (Fig 3E, right panel), whereas the double D320E/Y321N substitutions render the replicon (EC_50_ of 75 nM) 2.1-fold more resistant to STG-175 than wild-type replicon (EC_50_ of 35.7 nM). At day 8, the D320E substitution renders the replicon (EC_50_ of 83.2 nM) 2.4-fold more resistant to STG-175 than wild-type replicon (EC_50_ of 34.4 nM) (Fig 3E, right panel), whereas the double D320E/Y321N substitutions render the replicon (EC_50_ of 101.5 nM) 2.9-fold more resistant to STG-175 than wild-type replicon (EC_50_ of 34.4 nM). These data indicate that the NS5A D320E and Y321N substitutions render HCV only partially (~2-3-fold) resistant to STG-175.

### Cross-resistance studies

**Fig 5:**
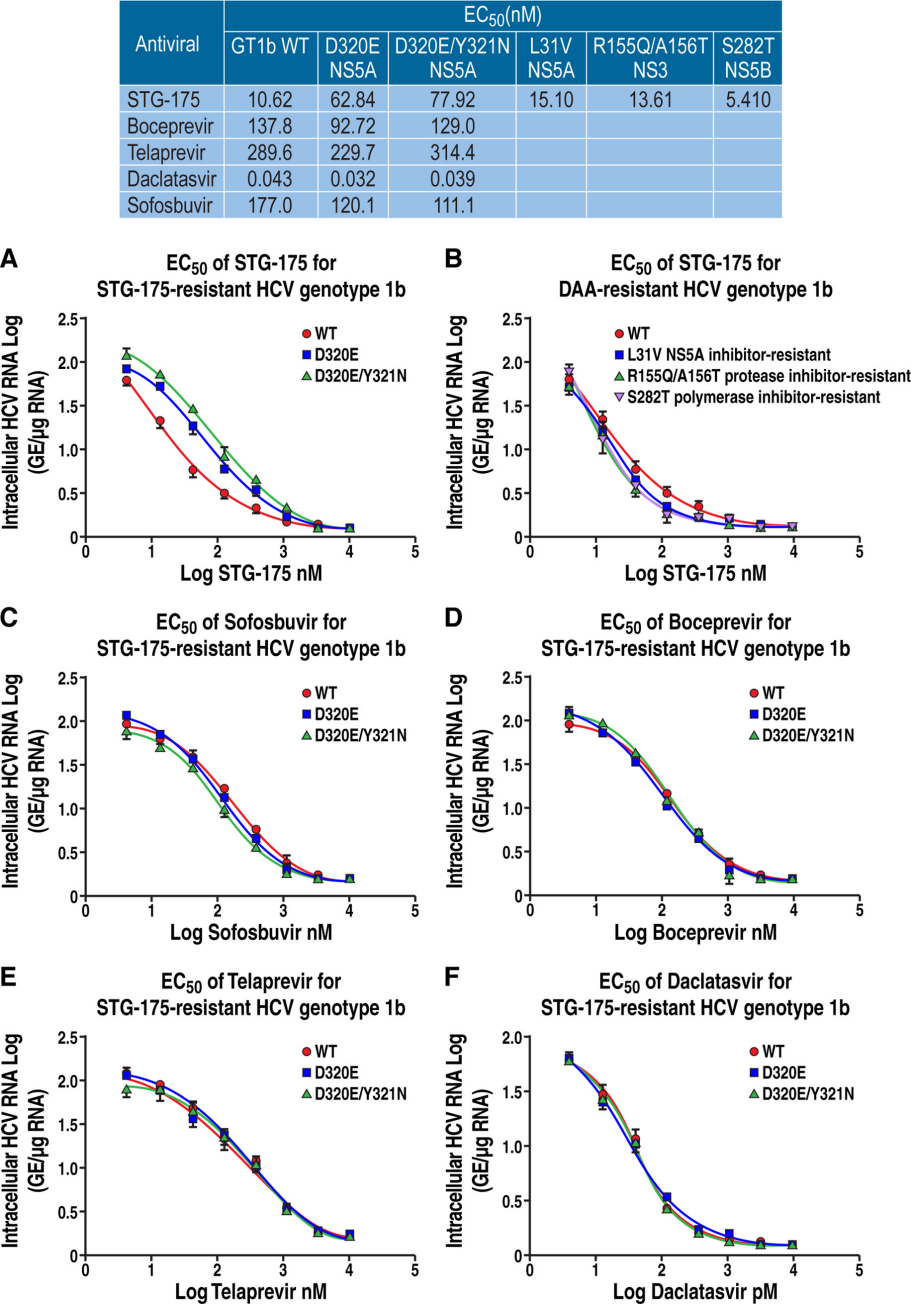
Cross-resistance analyses. Wild-type (WT), D320E and D320/Y321N NS5A Con1 RNA were electroporated into Huh7.5.1 cells in the presence of increasing concentration of anti-HCV agents: boceprevir (A), telaprevir (B), daclatasvir (C), sofosbuvir (D) and STG-175 (E), and levels of HCV RNA quantified 8 days post-electroporation. Data are expressed as log of GE/μg of total RNA. F. Same as A-F, except that cells were electroporated WT, NS5Ai-resistant (L31V), NS3i-resistant (R155Q/A156T) and NS5Bi-resistant (S282T) Con1 HCV RNA variants in the presence of increasing concentrations of SGT-175 and levels of HCV RNA quantified 8 days post-electroporation. Data were calculated as log of genome equivalents (GE)/μg of total RNA and the RNA amount was normalized to a range of 0–100% in order to calculate the EC_50_. Results are representative of two independent experiments. Data are representative of two independent experiments.

We then asked whether the two STG-175 partially (2-fold) resistant D320E and D320E/Y321N NS5A Con1 (GT1b) variants are resistant to DAAs. First, we quantified the resistance of D320E and D320E/Y321N NS5A Con1 variants to STG-175. We calculated EC_50_s of 10.62, 62.84 and 77.92 nM for wild-type, D320E and D320E/Y321N replicons, respectively (Fig 5A). These data indicate that the two partially STG-175-resistant D320E and D320E/Y321N variants remain fully sensitive to DAAs. We calculated EC_50_s i) for daclatasvir of 43, 32 and 39 pM for wild-type, D320E and D320E/Y321N replicons, respectively (Fig 5B); ii) for sofosbuvir of 177, 120.1 and 111.1 nM for wild-type, D320E and D320E/Y321N replicons, respectively (Fig 5C); iii) for boceprevir of 137.8, 92.72 and 129 nM for wild-type, D320E and D320E/Y321N replicons, respectively (Fig 5D); and iv) for telaprevir of 289.6, 229.7 and 314.4 nM for wild-type, D320E and D320E/Y321N replicons, respectively (Fig 5E). Thus, the two STG-175 partially resistant variants are sensitive to all tested DAAs.

We then asked whether DAA-resistant HCV variants are sensitive to STG-175. To address this inquiry, we took advantage of DAA-resistant Con1 (GT1b) variants, which we previously generated [21] including the NS3i-resistant R155Q/A156T, the NS5Bi-resistant S282T and the NS5Ai-resistant L31V variants. For STG-175, we calculated EC_50_s of 10.62, 15.1, 13.61 and 5.41 nM for wild-type, NS5A L31V, NS3 R155Q/A156T and NS5B S282T variants, respectively (Fig 5F). These data indicate that STG-175 remains efficient against DAA-resistant viruses. This further suggests that combining the CypI STG-175 with current DAAs is an attractive therapeutic option.

### Drug combination studies

**Fig 6:**
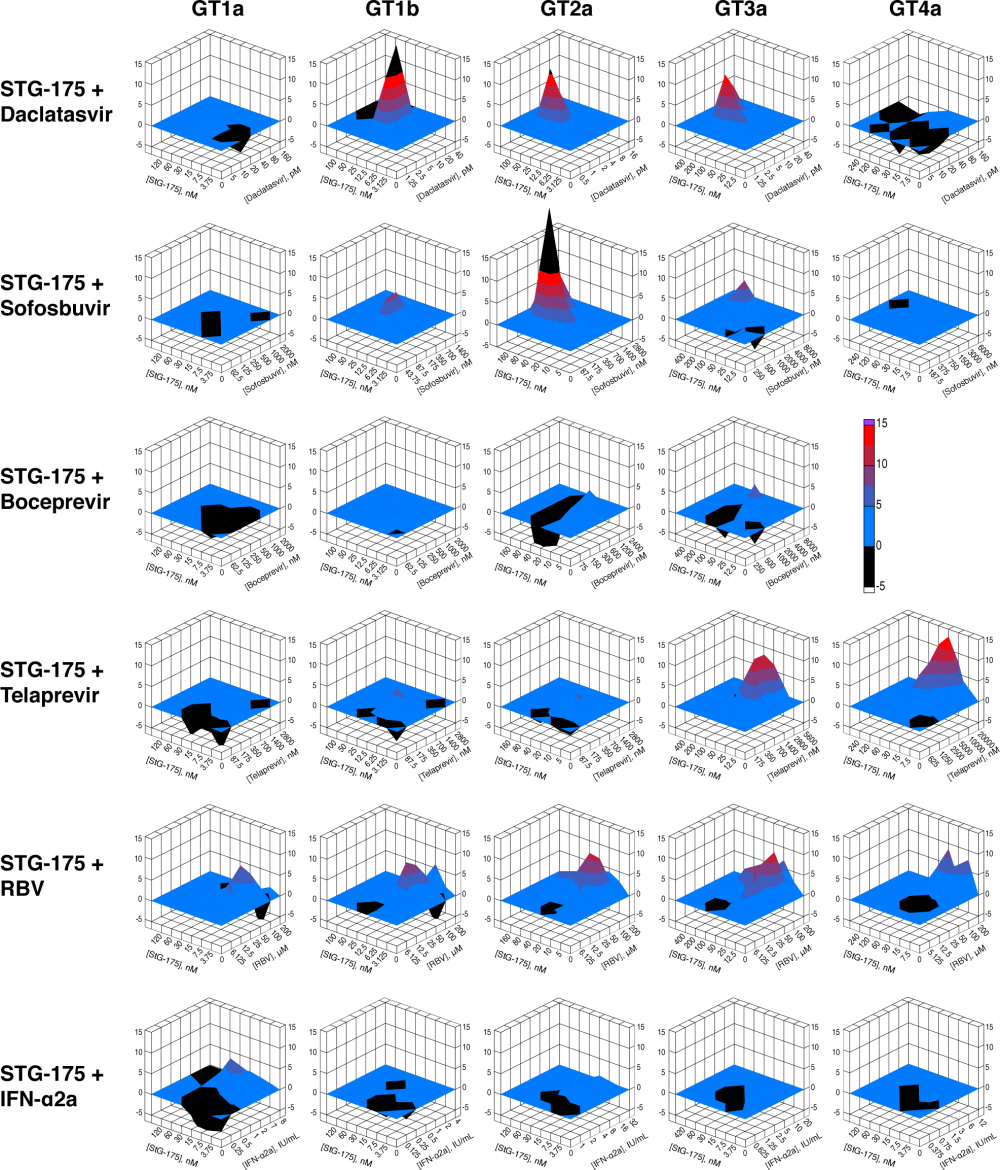
Drug combination analyses. Drugs were tested in pairs in 7 concentrations around calculated EC_50_ in each replicon cell lines (GT1-4). Cell viability and reporter assays were carried out as we described previously [21] in 5 replicates and normalized data was used to measure the additive, synergistic and antagonistic effect between drug combinations using the mathematical model MacSynergyII. The three-dimensional response surface plot represents the differences between actual experimental effects and theoretical additive effects at various concentrations of the two selected drugs. The colors are based on a scale (-5 to +15) used to represent additive, synergistic or antagonistic effects. The plane (0%), in blue, represents an additive effect of the two drugs tested in each plot. A peak over the 0% plane indicates synergy, higher the peak, greater the synergy. A trough, on the other hand, represents antagonism. The colors used, blue to red, indicate increasing synergy with red representing a higher synergy. A synergy beyond the chosen scale is represented by black. For each plot, the data was based on the 95% confidence interval for the dose-response values using MacSynergyII. Presented results are representative of two independent experiments.

After demonstrating no cross-resistance between STG-175 and DAAs, we conducted drug combination studies in order to determine the additive, synergistic and antagonistic effect of STG-175 combined with selected DAAs. We combined STG-175 with the two NS3i telaprevir and boceprevir, the NS5Ai daclatasvir, the NS5Bi sofobosvir, IFNα2a and RBV and tested their dual inhibitory activities on GT1a, GT1b, GT2a, GT3a and GT4a replicons. We did not observe significant antagonistic effects for any combinations between STG-175 and DAAs (Fig 6). For GT1a, we observed an additive effect for all drug combinations. For GT1b, we observed a synergistic effect for the STG-175/daclatasvir combination and additive effects for all other drug combinations. For GT2a, we observed a synergistic effect for the STG-175/daclatasvir, STG-175/sofosbuvir and STG-175/RBV combinations and additive effects for the other drug combinations. For GT3a, we observed a significant synergistic effect for the STG-175/daclatasvir, STG-175/telaprevir and STG-175/RBV combinations, a partial synergistic effect for the STG-175/sofosbuvir combination, and additive effects for the other drug combinations. For GT4a, we observed a significant synergistic effect for STG-175/telaprevir, a partial synergistic effect for STG-175/RBV, and additive effects for the other drug combinations. Since we found that boceprevir is inactive against GT4a [21], we did not test the STG-175/boceprevir combination on GT4a.

### Wide spectrum of antiviral activities of STG-175

**Fig 7:**
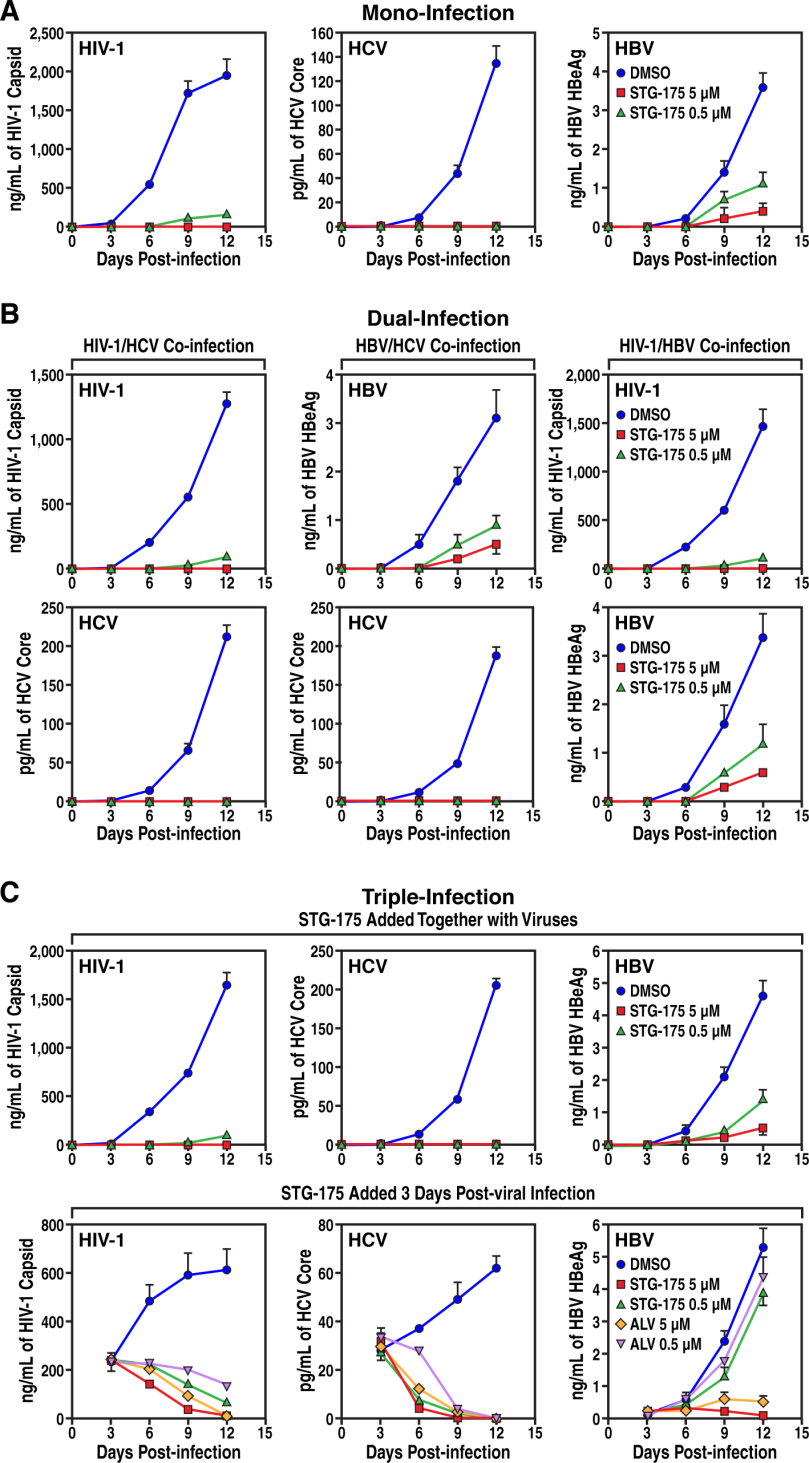
Mono- and co-infection analyses. A. *Mono-infection*. Left panel. Activated PBMCs were exposed to HIV-1 JR-CSF (TCID_50_ of 10^3^/mL) together with or without STG-175 for 3 h, washed and replication monitored by HIV-1 p24 ELISA for 12 days. Middle panel. Huh7.5.1 cells were exposed to HCV JFH-1 (TCID_50_ of 10^3^/mL) for 3 h, washed and replication monitored by HCV core ELISA for 12 days. Right panel. NTCP-Huh7 cells were exposed to HBV AD38 (TCID_50_ of 10^6^/mL) for 3 h, washed and replication monitored by HBV HBeAg ELISA for 12 days. B. *Dual-infection*. Left panels. Same as A, except that mixed PBMCs and Huh7.5.1 cells were exposed to both HIV-1 and HCV. Middle panels. Same as A, except that mixed NTCP-Huh7 and Huh7.5.1 cells were exposed to both HBV and HCV. Right panels. C. Same as A, except that mixed PBMC and NTCP-Huh7 cells were exposed to both HIV-1 and HBV. C. *Triple-infection*. Same as A, except that mixed PBMCs, NTCP-Huh7 and Huh7.5.1 cells were exposed to HIV-1, HBV and HCV altogether. DMSO, STG-175 or ALV was added to cells either together with viruses (top panels) or 3 days after virus addition and then every 3 days (bottom panels).

After demonstrating the high efficiency of STG-175 at blocking the replication of HCV of multi-GTs and clearing cells from HCV, we asked whether the CypI can also inhibit the infection of other prime human viruses. Specifically, we asked whether STG-175 decreases HIV-1 and HBV infection. Human activated PBMCs were exposed to HIV-1, NTCP-Huh7 cells were exposed to HBV, and Huh7.5.1 cells were exposed to HCV. DMSO or STG-175 (0.5 and 5 μM) was added together with viruses and replication monitored for 12 days by quantifying amounts of viruses in the supernatants of cell cultures by ELISA. We measured HIV-1 replication levels by HIV-1 capsid ELISA, HBV replication by HBV HBeAg ELISA, and HCV replication by HCV core ELISA. As expected, we found that STG-175 totally blocked HCV infection at both 0.5 and 5 μM (Fig 7A). At the high drug concentration (5 μM), STG-175 also fully blocked HIV-1 infection whereas at the lowest drug concentration (0.5 μM), it significantly decreased infection. Moreover, we found that STG-175 also significantly reduced HBV infection (Fig 7A). The degree of STG-175 antiviral efficacy was HCV > HIV-1 > HBV. Together these data indicate that the CypI STG-175 possesses a relatively wide spectrum of antiviral activities.

Since HCV patients are often co-infected with HIV-1 and HBV, we asked whether STG-175 could block a dual or a triple infection. To test this hypothesis, we cultured together (same flask) the three target cell populations for HIV-1, HBV and HCV: activated PBMCs, NTCP-Huh7 cells and Huh7.5.1 cells, respectively. A combination of two (dual infection) or three (triple infection) viruses was added to the cell co-cultures and replication of the three viruses monitored by quantifying viral levels in supernatants by ELISA as above. DMSO and STG-175 (0.5 and 5 μM) were added either together with the viruses or 3 days post-infection followed by drug addition every 3 days. As for the mono-infections (Fig 7A), STG-175 inhibited in a dose-dependent manner dual (Fig 7B) and triple (Fig 7C) HIV-1/HCV/HBV co-infections. The addition of STG-175 together with virus totally blocked HCV and HIV-1 infection and greatly attenuated HBV infection. When added 3 days post-infection and then every 3 days, STG-175 totally eradicated the pre-established HCV infection, almost totally aborted the established HIV-1 infection and decreased the viral expansion of the pre-established HBV infection (Fig 7C).

## Discussion

In this study, we investigated the anti-HCV properties of a new CypI—STG-175. We demonstrated that STG-175 possesses high anti-PPIase activities against CypA and CypD. The anti-isomerase efficiency of STG-175 was superior to that of other CypI including CsA, SCY-635 and ALV. We found that STG-175 is deprived of immunosuppressive activities compared to the immunosuppressive CypI—CsA. We showed that STG-175 has a multi-genotypic activity, exhibits a 13–39 nM EC_50_ range among HCV GTs, and does not significantly lose its antiviral activity in the presence of high concentration of human serum. Since we and others showed that host CypA is indispensible for the replication of all HCV GTs [35, 37–42], one can speculate that the multi-genotypic activity of STG-175 is due to the fact that it neutralizes the isomerase activity of CypA (Fig 1B).

We found that STG-175 at a concentration as low as 250 nM cures cells of HCV and prevents viral replication rebound. We also found that STG-175 presents a high barrier to resistance since many cell culture passages (>16 passages) were required to detect the emergence of replicon colonies growing even in the presence of the drug. STG-175 presented a higher barrier to resistance than other CypI such as CsA and ALV as well as DAAs such as telaprevir, boceprevir and daclatasvir. Two mutations—D320E and Y321N - were identified in the NS5A region of the STG-175-resistant replicon colonies. The D320E and Y321N mutations have been previously identified in HCV variants resistant to other CypI including CsA, ALV, SCY-635 and NIM811 [43–49]. Thus, it is likely that the double E320/N321 substitutions represent a common motif of partial resistance to CypI including STG-175. Our finding that a single and a double NS5A substitution emerged under the low (0.25 μM) and high (1 μM) STG-175 concentration, respectively, further suggests as we previously reported [45] that multiple mutations in HCV NS5A domain II may confer some resistance to ALV (2-3-fold). Note that the resistance of the colonies (A to E), which emerged after multiple passages under 0.25 and 1 μM of STG-125, is highly superior to that of “cloned” D320E and D320E/Y321N replicons. Specifically, colonies A, B and C (with a D320E substitution) exhibited an average 10-fold higher resistance to STG-175 than wild-type colonies (157.8 nM versus 15.7 nM), whereas colonies D and E (with the double D320E/Y321N substitutions) exhibited an average 33.8-fold higher resistance to STG-175 than wild-type colonies (531 nM versus 15.7 nM). In contrast, we found that the introduction of the single D320E substitution and double D320E/Y321N substitutions into wild-type replicon render the replicon only moderately resistant to STG-175. Specifically, the D320E and D320E/Y321N replicons exhibited a 1.7-fold and 2.1-fold higher resistance to STG-175 than wild-type replicon. Since we sequenced the HCV subgenome of the five colonies (A to E) and identified substitutions exclusively in NS5A, the higher resistance of the colonies than that of “cloned” D320E and D320E/Y321N replicons should originate from a non-viral component. One can envision that cells after many passages under STG-175 selection became “adapted” to the drug exposure. After weeks/months of drug treatment, cells eventually “evolved” to either degrade STG-175 more rapidly, to bind STG-175 via host proteins other than CypA or to increase intracellular levels of CypA. This could eventually explain why the STG-175-resistant colonies (A to E) are more resistant to the drug than the “cloned” D320E and D320E/Y321N replicons. It is important to note that to date that in patients no correlation was observed between ALV resistance and specific mutations in the HCV genome [50]. Together these observations strongly suggest that CypI such as STG-175 should present a high barrier of resistance both *in vitro* and *in vivo*. STG-175 is more efficient than ALV and CsA at inhibiting HCV replicon replication and at clearing replicon replication and preventing viral replication rebound likely due to its superior ability to neutralize the isomerase activity of CypA (EC_50_ = 0.6 nM) than that of ALV (1.7 nM) and CsA (11.3 nM) (Fig 1A).

No cross-resistance was observed between STG-175-resistant and DAA-resistant HCV variants. This is consistent with the fact that CypI have a unique MoA, which is the prevention of contacts between the host protein CypA and the viral protein NS5A [38, 43–47, 51–53]. We showed that CypI such as ALV and SCY-635 block interactions between CypA and NS5A derived from various GTs [43–44]. The Bartenschlager lab who pioneered these captivating findings [54] as well as more recently our lab [55–56] demonstrated that CypI such as cyclosporine D (CsD), ALV and CPI-431-32 prevent the formation of double membrane vesicles (DMVs) in HCV-infected cells. HCV is well known to reorganize the intracellular membranes to optimize its replication. DMVs serve as membranous compartment where HCV replication occurs efficiently and safely, protected from cellular RNA sensors and degradation factors [57–59]. Interestingly, the Bartenschlager lab and others provided evidence that NS5Ai, like CypI, also prevent the formation of DMVs [55, 60–61]. Importantly, we showed that excepted CypI and NS5Ai, no other classes of anti-HCV agents inhibit the formation of DMVs by HCV [55]. The recent findings that both CypI and NS5Ai mediate this block, strongly suggest that CypA and NS5A act in concert to form these protective organelles in HCV-infected cells. Further work is required to unravel the precise MoA of CypA and NS5A (or other additional host and viral proteins) in this process.

Our drug combination studies suggested that the combination of STG-175 with the NS3i telaprevir and boceprevir, the NS5Ai daclatasvir, the NS5Bi sofosbuvir, IFNα2a and RBV on GT1a, GT1b, GT2a, GT3a and GT4a exert no significant antagonistic effect for any STG-175 combinations on any GTs. In general, we observed an additive effect for all STG-175 combinations throughout all GTs. Importantly, we observed a significant synergistic effect for GT1b, GT2a and GT3a when STG-175 was combined with daclatasvir. We also observed a significant synergistic effect for GT2a and a partial synergistic effect for GT3a when STG-175 was combined with sofosbuvir. This is important since daclatasvir and sofosbuvir are currently used with success in HCV patients in IFN-free treatments. These results are similar to those obtained for the other CypI—ALV. Specifically, we observed a partial and significant synergistic effect on GT2a and GT3a, respectively, when we combined ALV and daclatasvir, and a partial and significant synergistic effect on GT3a and GT2a, respectively, when we combined ALV and sofosbuvir [21]. The synergistic effect of the STG-175/daclatasvir combination could arise from the fact that both drugs–the cyclophilin inhibitor STG-175 and the NS5A inhibitor daclatasvir–target indirectly (STG-175) and directly (daclatasvir) NS5A. Indeed, on one hand cyclophilin A binds to the domain II of NS5A, and cyclophilin inhibitors block this contact [51], while NS5A inhibitors neutralize NS5A action by binding to the domain I of NS5A. Thus, the STG-175/daclatasvir combination may exert its synergistic effect by targeting two distinct domains and/or actions of NS5A. We previously proposed a similar synergistic model for the ALV/daclatasvir combination [21]. The synergistic effect of the STG-175/sofosbuvir combination is more difficult to explain except that it could arise from the fact that cyclophilin A and NS5B binds to the same region of NS5A [62]. Thus, cyclophilin inhibitors such as STG-175 by preventing cyclophilin A-NS5A contacts would allow NS5B to interact with NS5A. One could envision that when the polymerase NS5B is bound to NS5A, its enzymatic activity is more sensitive to an inhibitory action of polymerase inhibitors such as sofosbuvir.

Our co-infection (co-culture) studies reveal the ability of STG-175 at inhibiting three concurrent infections and replications: HCV, HIV-1 and HBV. We found the following degree of STG-175 antiviral efficacy: HCV > HIV-1 > HBV. These differences in the degree of sensitivity to STG-175 between the three viruses may originate from either different MoA of CypI or different degrees in the requirement for CypA in the three viral life cycles. The MoA of CypI for the three viruses are distinct and remain poorly understood. For HCV, the current model is that CypI, by blocking HCV NS5A-CypA interactions, prevent DMVs formation and thus HCV RNA replication. For HIV-1, the current model is that CypI, by blocking HIV-1 capsid-CypA interactions, prevent the nuclear import of the viral genome and its subsequent integration into the host chromosomes. For HBV, the current model is that CypI exert two independent inhibitory actions: i) an entry block, by binding to NTCP and preventing the viral glycoprotein from interacting with this cell surface receptor [63–64]; and ii) a still unknown post-entry block, likely at a transcription or translation step since CypI such as ALV inhibits HBsAg production upon HBV transfection [65]. Further work is required to fully elucidate the antiviral MoA of CypI for these three viruses. A difference in the degree of requirement for CypA in the three viral life cycles may also explain the differences in the degree of sensitivity to STG-175 among the three viruses. We and others showed that knocking down CypA by shRNA or siRNA in target cells totally inhibits HCV infection [37–38, 40–41] while it partially decreases HIV-1 [66–68] and HBV [65] infection. Thus, HCV highly relies on CypA to replicate while HIV-1 and HBV requires CypA to optimally replicate in target cells. These data are consistent with our findings that CypI inhibit HCV replication at a nM range whereas they inhibit HIV-1 and HBV replication at a μM range.

In conclusion, by demonstrating a wide spectrum of antiviral activity, a multi-genotypic anti-HCV activity, an additive to synergistic effect when combined with specific DAAs, and a high barrier to resistance—the new CypI STG-175 represents an attractive drug partner in IFN-free regimens for the treatment of HCV and co-infections.
